# Coupling Coordination and Spatiotemporal Dynamic Evolution Between Medical Services and Tourism Development in China

**DOI:** 10.3389/fpubh.2022.731251

**Published:** 2022-01-31

**Authors:** Shaogui Xu, Yifan Zuo, Rob Law, Mu Zhang, Jiayu Han, Gaopeng Li, Juewei Meng

**Affiliations:** ^1^School of Management, Jinan University, Guangzhou, China; ^2^Shenzhen Tourism College, Jinan University, Shenzhen, China; ^3^Department of Integrated Resort and Tourism Management, Faculty of Business Administration, Asia-Pacific Academy of Economics and Management, University of Macau, Macau, China; ^4^Graduate School of Guangxi Medical University, Nanning, China; ^5^School of Acupuncture and Massage, Guangxi University of Chinese Medicine, Nanning, China

**Keywords:** tourism development, medical services, coupling coordination degree model (CCDM), spatiotemporal dynamic evolution, trend surface analysis, geodetector, China

## Abstract

This work constructs an evaluation index system and quantitatively explores the coupling coordination relationship between the tourism development system and the medical services system in China. Results show that the degree of coupling coordination between the tourism development system and the medical services system showed a good upward trend in China during the period 2012–2019. However, the relationship was barely balanced, with tourism development lagging. The overall layout shows a spatial pattern of “high in the north and low in the south, high in the east and low in the west.” The degree of coupling coordination tends to be randomly distributed from clustered distribution, and the cold–hot spots show a spatial development pattern of “cold in the northwest and hot in the southeast” as time passes. The power of government to regulate has always been an important mechanism affecting the degree of coupling coordination. The study aims to provide reference for the rationalization of medical tourism layout and sustainable development.

## Introduction

From ancient times to the present, a healthy body has been one of the primary demands of human beings at every stage of individual human development. The need for wellness and beauty has become commonplace with the development of society; hence, medical tourism has attracted widespread attention as a growing number of people from high-income countries travel to low- and middle-income countries for healthcare (1, 2). Although medical tourism is multi-directional and complex, and no reliable verifiable statistics are available (3, 4), relevant information shows a number of patients traveling to developing countries for healthcare, and medical tourism has good prospects for growth (5, 6).

Medical tourism is not a new phenomenon, and it is rapidly growing across the world; however, it has no single definition (2, 4). Some scholars consider medical tourism as a subset of health tourism or conflate them (7, 8). Meanwhile, most scholars attempt to distinguish medical tourism from health tourism. Connell (9) argues that medical tourism is difficult and often futile to define. Nonetheless, the common characteristics can be distinguished (2). For example, medical tourism mainly refers to tourists who undertake tourism activities across national borders and choose their destinations according to their own intentions, unlike general tourism products (4, 10, 11). On the basis of these characteristics, this work considers medical tourism as a phenomenon in which travelers cross borders, choose their own destinations, and aim to access medical resources and services (5, 12). Connell (10) argues that international medical tourism has evolved through three stages. Initially, developed countries had advanced medical technology and standards, thereby attracting wealthy medical tourists from other developing countries to travel for medical treatment. In the second stage, many tourists from developed countries were attracted to seek medical treatment in these countries as developing countries improved their medical technology and tapped into traditional medical techniques, coupled with relatively low medical costs. In the third stage, medical tourists from developed and developing countries traveled to each other's countries (10). The international medical tourism market is currently at the third stage.

At present, the demand for medical tourism is rapidly increasing due to aging, sub-health, and rising health-care cost. In the emerging field of medical tourism, many countries see significant economic development potential. Asia-Pacific is the fastest growing and dominant region in the international medical tourism field (13, 14). Despite the late start of medical tourism in China, the country is rich in Chinese medicine resources, the national health awareness has increased, and the Chinese medical sector has unique advantages in developing medical tourism with its traditional techniques and the broad market. Moreover, the Chinese government attaches great importance to the development of medical tourism. In 2013, China's State Council proposed to “develop health culture and tourism and encourage regions with the conditions to develop health, sports, and medical tourism for the international and domestic markets” and formally approved the establishment of the Boao Lecheng International Medical Tourism Pilot Zone in Hainan. In 2016, the “Health China 2030” plan was released, and it was proposed to actively promote the integration of health with elderly care, tourism, the Internet, fitness and leisure, and food. However, medical tourism products specifically for foreign tourists are rare, and the number of foreign tourists visiting China to receive medical services is small. In terms of inbound medical tourism, China lags considerably behind Thailand, Singapore, and India (15, 16).

Research on medical tourism has become a hot topic. Academics have shown considerable interest in medical services and tourism development. They argue that medical tourism is a “combination of medical services and tourism development” (6, 17). Despite a high degree of fit between medical services and tourism development, understanding exactly how they are coupled is a challenging task. Given that the coupling is considerably complex to measure, no scientifically sound evaluation system has been created. Existing studies rarely consider the coupling between the two and neglect to use geographic methods to visualize the spatial characteristics and dynamic evolution of the coupling coordination. Although the medical tourism in China has great potential, research has started late and lacks a mature theoretical framework to guide medical tourism practice (15, 16, 18). Therefore, an in-depth study of the coupling relationship between medical services and tourism development in China is needed.

This study first establishes the indicator system of medical services and tourism development. Then, a comprehensive level evaluation of medical services and tourism development in 31 provinces of China between 2012 and 2019 was conducted, and the coupling coordination degree model (CCDM) was used to gain an accurate insight into the coupling effect between medical services and tourism development. Finally, the spatial–temporal evolution of the coupled and coordinated relationship between the two systems is analyzed. In summary, this work aims to: (1) reveal the comprehensive development level of medical services and tourism development; (2) analyze the development trend of coupling and coordination between medical services and tourism development through CCDM; (3) examine the spatial and temporal evolution patterns and characteristics of the degree of coupling coordination; and (4) reveal the driving mechanism of the degree of coupling coordination through a geodetector. The study will provide a theoretical basis for the development of medical tourism in China.

## Literature Review

### Effect of Tourism Development on Medical Services

The growth of the tourism industry has stimulated governments to expand investment in medical services. Over the past decade, many countries have identified tourism as a pillar industry that can generate foreign exchange earnings, boost economic growth, increase tax revenues, and create a large number of jobs (19). Tourism spurs the development of sectors, such as retail, hospitality, transport, and infrastructure, and stimulates the growth of local health industries (20, 21). Moreover, it will promote host countries' export services, creating good conditions for building a good image of the country and for the development of medical and tourism businesses (1, 20, 22). Accordingly, governments provide a range of subsidies and fiscal incentives to encourage investors and promote the development of healthcare services, such as policies for tax breaks, financial support for equipment, provision of land for healthcare services, and support for overseas healthcare investment (23). In particular, developing countries have attempted to improve the professionalism and maturity of the local healthcare sector by using the latest systems and equipment (24).

Tourism development contributes to the optimization of medical services. In a market where tourism demand tends to be personalized and diversified, people traveling to overseas countries will want to access medical services, such as dental, cosmetic, fertility, and surgery (10, 25). To attract foreign patients, private healthcare providers make significant investments to upgrade their healthcare facilities and train and attract highly skilled healthcare workers (20). Thus, tourism not only contributes to the development of hospitality and transportation but also improves the accessibility and quality of healthcare in many countries (especially developing countries) and can effectively stimulate healthcare providers to provide better quality healthcare services (26), thereby helping in extending the chain and strengthening the link between tourism and healthcare industries (27).

However, tourism development can create inequalities in medical services. The development of tourism has led to significant economic gains and job creation in the health sector for residents of tourist destinations, further increasing the social well-being of residents and improving the quality of life (28). However, the rapid growth of tourism can create conflicts between a large number of tourists and the local population in terms of their healthcare needs. For example, medical tourism could lead to a reduction in access to medical services for the local population, which could have a negative effect on health equity (20). It may lead to the transformation of traditional healthcare services enjoyed by residents into commercial opportunism, such as tourist congestion and high costs of healthcare services (9). A growing patron-client conflict between residents and tourists may also ensue (19). Legal and ethical issues may also arise (29, 30).

### Effect of Medical Services on Tourism Development

Medical services are encouraging countries to increase investment in tourism. In the broader context of the global healthcare industry, the trends of an aging population, increasing health problems, and rising healthcare expenditure place great pressure on patients (31, 32). Accordingly, many patients from developed countries travel abroad to seek low-priced and quality healthcare services (33). Many developing countries are actively investing in the tourism industry, increasing tourism infrastructure, and improving public service support facilities to retain large numbers of tourists and generate objective foreign exchange earnings from tourism (34).

Medical services help increase tourism revenues. The high quality of medical services in a destination country, the effect of branding, and the government's cooperation strategy greatly enhance the attractiveness of a destination, thereby increasing the number of inbound tourists, which, in turn, increases the local tourism revenue (14, 35). Stimulated by the huge foreign exchange earnings, the government aims to promote local tourism development by simplifying visa applications, developing transportation, improving tourism infrastructure, and promoting investment (9, 35).

However, medical services can affect tourism development. Controversies arise over sensitive medical issues, such as human organ transplants, euthanasia, and abortion, because of the differences in laws and ethics in different countries. From a risk perspective, medical safety incidents are an important influencing factor. For example, when a patient has completed treatment and is likely to travel to another country or return home, continuing treatment or securing legal rights in the event of relapse become difficult, which can increase a patient's medical costs (36, 37).

### Relationship Between Tourism Development and Medical Services

The relationship between medical services and tourism development is complex because they have their structures, functions, and development rules and are also constrained by each other (38). The development of tourism provides a medium for the promotion of medical services, and the improvement of the level of medical services causes a tourism product to become diversified and better suited to the needs of tourists. A positive relationship (or negative relationship) can be observed between the two systems (39). The degree of coupling refers to the strength of the interaction between the two parties, regardless of the benefits and drawbacks. The degree of coordination refers to the magnitude of benign coupling in the interaction, reflecting a good or bad coordination status, and can characterize whether the functions promote each other at a high level or constrain each other at a low level (40, 41). Coupled coordination analysis refers to the quantitative analysis of the degree of association between two or more systems and can be used to measure the tightness of the relationship between systems (42, 43).

At present, researchers have used this approach to study the relationship between tourism and economy (44, 45), tourism and culture (46), tourism and environment (43, 47), urbanization and environment (48), economic development and ecology (49), and urbanization and geo-hazards (39). These related studies reveal the correlated roles between two systems and indicate that they dynamically change over time (50). Although coupled coordination has been widely applied in the tourism sector, no studies using this method have explored the interactions between medical services and tourism development. Therefore, this study focuses on the spatial–temporal correlation and heterogeneity between the development of medical services and tourism in China to fill the gaps in existing research. The possible causes of conflicts between medical services and tourism development can be identified by analyzing their degree of coupling and coordination, which will help in the promotion of their coordinated development and contribute to the sustainable development of medical tourism.

## Materials and Methodology

### Indicator System

An extremely complex interaction exists in the relationship between medical services and tourism development. Tourism development promotes the growth of medical services, whereas medical services support the tourism development. These two aspects complement each other in a dynamic process. Tourism development has a huge tourism multiplier effect on medical services, which not only brings major economic benefits and creates employment opportunities but also drives the development of upstream and downstream industries and extend the medical services industry chain (20, 21). However, it can also create social problems, such as inequality in healthcare services (20). Correspondingly, medical services also greatly support the development of the tourism industry.

**Figure 1:**
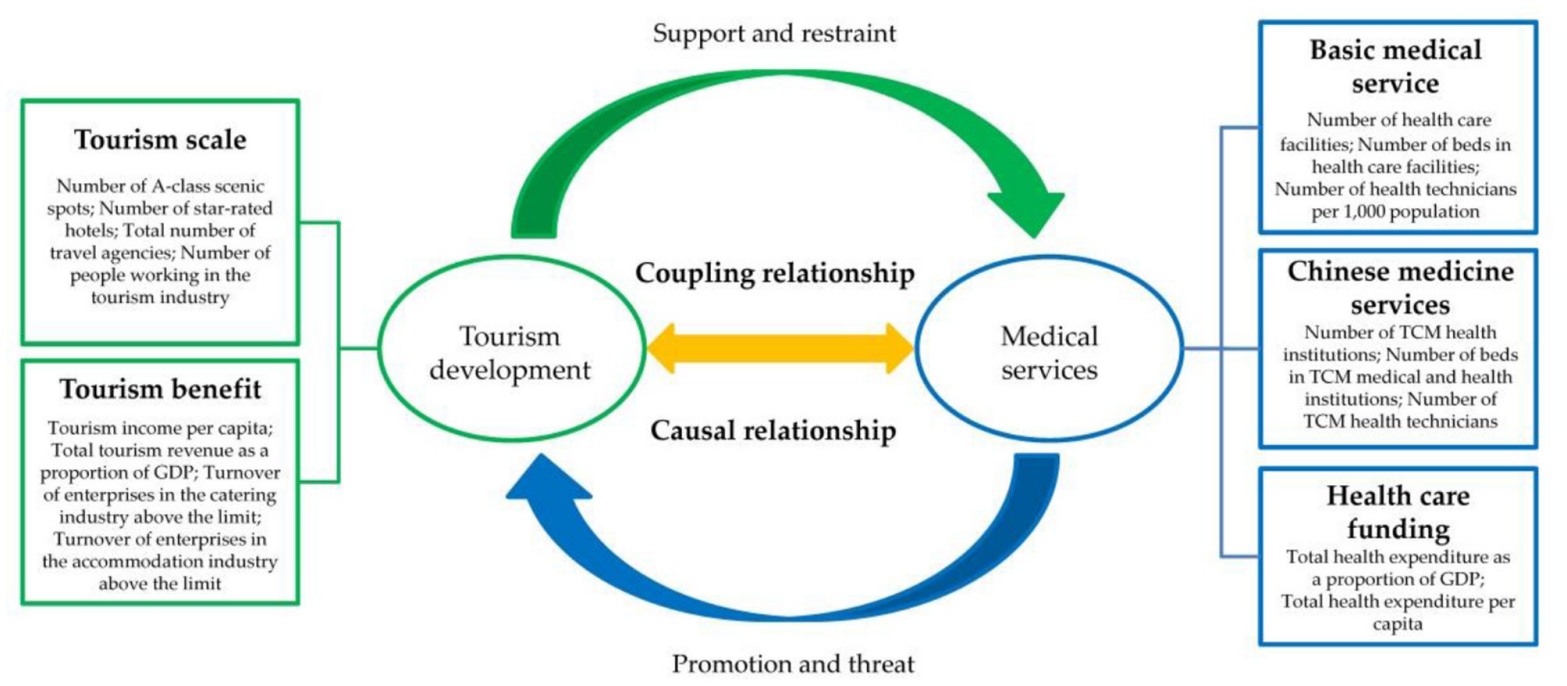
Conceptual framework for the relationship between tourism development and medical services.

Medical services provide professional technical support to the tourism industry, enriching the range of tourism products, improving the quality of services, and promoting the upgrade and optimization of the tourism industry structure (9, 14, 35). However, the health services sector can also have an influence on tourism (36, 37). Accordingly, the two aspects are causally related, interdependent, and indispensable. Given that tourism is an integrated industry, it is closely related to other industries and social services (44). Therefore, a system consisting of a tourism development system and a healthcare service system can be defined as a coupled and coordinated system, and the coordination of the coupling is the basis for achieving sustainable development of medical tourism (Figure 1).

We constructed a comprehensive evaluation indicator system using existing indicators to measure the close relationship between tourism development and medical services. The selection of indicators was based on these principles: first, the most frequently cited indicators were chosen; second, the components of tourism development and healthcare services were included; and third, the indicators chosen were of good typicality and practicality. Indicators for tourism development systems were initially identified according to previous studies (43, 44, 51, 52). Indicators for the healthcare delivery system were then further selected through analysis of relevant literature and in the context of China-specific situations to gain insight into the coupling between tourism development and healthcare delivery and identify the interactions between these factors. Finally, a comprehensive evaluation indicator system, consisting of 2 systems, 5 subsystems, and 16 indicators, was constructed (Table 1).

**Table 1:** Coupling coordinated evaluation index system of tourism development and medical services.

**Systems**	**Subsystems**	**Indicators**	**Units**	**Weight**
Tourism development	Tourism scale	Number of A-class scenic spots	Unit	0.111
		Number of star-rated hotels	Unit	0.097
		Total number of travel agencies	Unit	0.104
		Number of people working in the tourism industry	Person	0.114
	Tourism benefit	Tourism income per capita	Million yuan	0.095
		Total tourism revenue as a proportion of GDP	%	0.070
		Turnover of enterprises in the catering industry above the limit	Billion yuan	0.232
		Turnover of enterprises in the accommodation industry above the limit	Billion yuan	0.178
Medical services	Basic medical service	Number of health care facilities	Unit	0.156
		Number of beds in health care facilities	Beds	0.108
		Number of health technicians	Thousand person	0.113
	Chinese medicine services	Number of TCM health institutions	Unit	0.126
		Number of beds in TCM medical and health institutions	Beds	0.101
		Number of TCM health technicians	Person	0.116
	Health care funding	Total health expenditure as a proportion of GDP	%	0.115
		Total health expenditure per capita	Yuan	0.165

According to industrial development theory, the tourism development system is a complex system whose industrial development is driven by a variety of factors, and the tourism development system evaluation index system can be examined from two subsystems (43, 44, 53). The first subsystem is the scale of tourism. It reflects the overall level of the tourism industry. The number of A-class scenic spots is used to reflect the endowment value and supporting capacity of tourism resources (51, 54). The number of star-rated hotels reflects the overall level of the tourism industry (54, 55). The total number of travel agencies reflects the service capacity of the tourism industry (53, 56). Moreover, the number of people working in the tourism industry reflects the tourism labor inputs (53, 57). The second subsystem is tourism benefit, which reflects the economic benefits and status of the tourism industry. The tourism income per capita reflects the economic effect of tourism (43, 51). The total tourism revenue as a proportion of GDP reflects the position of tourism in the national economy (53). The turnover of enterprises in the catering industry above the limit reflects the share of the catering sector in the tourism revenue structure (52). Moreover, the turnover of enterprises in the accommodation industry above the limit reflects the share of the accommodation sector in the tourism revenue structure (43, 52).

China's healthcare delivery system operates on a dual-track basis, including the public and private sectors. Public healthcare institutions are the mainstay and cornerstone of the healthcare delivery system (58). Chinese medicine and Western medicine co-exist in the current health care delivery system. Accordingly, this study considers three subsystems of the healthcare delivery system. The first subsystem is primary care services. The content of primary healthcare services mainly includes various institutions and facilities of disease treatment, convalescence and recuperation, treatment and examination, and the corresponding consumption of medicines. Thus, we measure the conditions for the development of basic medical services in terms of institutions, facilities, and personnel, which mainly include the number of health care facilities (24), the number of beds in health care facilities (14), and the number of health technicians (59, 60). The second subsystem is Chinese medicine services. Traditional Chinese medicine (TCM) not only plays an important role in the Chinese healthcare system, but it is also welcomed and respected worldwide in promoting health, well-being, and longevity. In particular, TCM has significantly contributed to the treatment of COVID-19 (61, 62). Chinese medicine services also measure the conditions for the development of Chinese medicine services in terms of institutions, facilities, and personnel, including the number of TCM health institutions (62), number of beds in health care facilities (61), and the number of TCM health technicians (58, 63). The third subsystem is health care funding, which mainly reflects the government's financial investment in health care services and the extent to which the government and society as a whole attach importance to the health of the population. Therefore, the evaluation indicators of health care funding include total health expenditure as a proportion of GDP (20, 60) and the total health expenditure per capita (64).

### Data Collection

The latest data available for this study are from 2019 because of the lag in the publication of statistics in China. Accordingly, we choose the period 2012–2019 as the study period and took 31 provinces (cities and districts) in China (excluding Hong Kong, Macao, and Taiwan) as the research object. The data used are mainly from the China Statistical Yearbook, the China Health Care Statistical Yearbook, and the statistical bulletin on the national economic and social development of each province.

### Methods

#### Integrated Development Level Evaluation Model (IDLEM)

The medical service system and the tourism development system are two systems that influence each other, and the level of their combined development needs to be measured. The calculation formula is as follows:


(1) $$\begin{array}{r} u_{i}=\overset{m}{\underset{j=1}{\sum }}\lambda_{ij}u_{ij},\;\overset{m}{\underset{j=1}{\sum }}\lambda_{ij}=1 \end{array}$$

where *u*_*i*_ refers to the comprehensive evaluation value of the system in year *i*, *u*_*ij*_ is the degree of contribution of indicator *j* to the system, and λ_*ij*_ refers to the weight of the indicator. The degree of contribution *u*_*ij*_ is calculated according to the effect of the indicator on the system using a positive efficacy function, as follows: *u*_*ij*_ = (*X*_*ij*_ − *X*_*jmin*_)/(*X*_*jmax*_ − *X*_*jmin*_), where *X*_*jmax*_ and *X*_*jmin*_ are the maximum and minimum values of the *j*th indicator, respectively. The indicator weights λ_*ij*_ are calculated by using the entropy method, which is objective and scientific. The entropy method can judge the degree of dispersion of indicators based on the size of information provided by the observations of each indicator and assign the indicator weights (43). First, the data must be standardized or normalized. The standardization of positive and negative indicators is distinguished because the magnitudes of the data used are different; second, the entropy value of each indicator is calculated; again, the weights of each indicator are determined according to the entropy value of each indicator (65–67). The entropy method is general, and the calculation steps are not repeated (68, 69). Table 1 shows the weights of each indicator.

### CCDM

The coupling equation in physics is used to model the degree of coupling between the medical services system and the tourism development system:


(2) $$\begin{array}{r} C=2\sqrt{\frac{q_{1}\times q_{2}}{{\left(q_{1}+q_{2}\right)}^{2}}} \end{array}$$

where *C* is the degree of coupling of the two systems. Relying solely on the degree of coupling is not possible because the coupling model can give the illusion that the degree of coupling is high when the comprehensive evaluation index of the system is low. Therefore, the coupling coordination model is constructed as follows:


(3) $$\begin{array}{r} D=\sqrt{CT} \end{array}$$

(4) $$\begin{array}{r} T=\alpha q_{1}+\beta q_{2} \end{array}$$

where *D* is the coupling coordination degree; *T* is the comprehensive evaluation index; and α and β are the weights of the medical services system and the tourism development system, respectively. Weights α and β are assigned to 0.5 by referring to related studies (40, 70); the higher the value of the degree of coupling coordination, the better the match between the two systems, and the system tends to develop in an orderly direction. In addition, the degree of coupling coordination and the comprehensive development level were ranked according to related studies to determine their development stages (39, 69) (Table 2).

**Table 2:** Classification standard of coupling coordination types between tourism development (TD) and medical services (MS).

**CCD**	**Stages of coupling development**	**Relationship between q_1_ and q_2_**	**Development modes between systems**
0.8 < *D* ≤ 1	Superiorly balanced development	*q*_1_ > *q*_2_	Superiorly balanced development with MS lagged
		*q*_1_ = *q*_2_	Superiorly balanced development between TD and MS
		*q*_1_ < *q*_2_	Superiorly balanced development with TD lagged
0.6 < *D* ≤ 0.8	Favorably balanced development	*q*_1_ > *q*_2_	Favorably balanced development with MS lagged
		*q*_1_ = *q*_2_	Favorably balanced development between TD and MS
		*q*_1_ < *q*_2_	Favorably balanced development with TD lagged
0.5 < *D* ≤ 0.6	Barely balanced development	*q*_1_ > *q*_2_	Barely balanced development with MS lagged
		*q*_1_ = *q*_2_	Barely balanced development between TD and MS
		*q*_1_ < *q*_2_	Barely balanced development with TD lagged
0.4 < *D* ≤ 0.5	Slightly imbalanced development	*q*_1_ > *q*_2_	Slightly imbalanced development with MS lagged
		*q*_1_ = *q*_2_	Slightly imbalanced development between TD and MS
		*q*_1_ < *q*_2_	Slightly imbalanced development with TD lagged
0.2 < *D* ≤ 0.4	Moderately imbalanced development	*q*_1_ > *q*_2_	Moderately imbalanced development with MS lagged
		*q*_1_ = *q*_2_	Moderately imbalanced development between TD and MS
		*q*_1_ < *q*_2_	Moderately imbalanced development with TD lagged
0 < *D* ≤ 0.2	Seriously imbalanced development	*q*_1_ > *q*_2_	Seriously imbalanced development with MS lagged
		*q*_1_ = *q*_2_	Seriously imbalanced development between TD and MS
		*q*_1_ < *q*_2_	Seriously imbalanced development with TD lagged

### Trend Surface Analysis

Trend surface is a semi-quantitative study of geographic data with a large spatial span based on spatial data and mathematical surfaces emulated by mathematical fitting, which can be used to explore the spatial trends and distribution patterns of geographic element observations (71, 72). In this work, we simulate the spatial and temporal variation characteristics of the coupling coordination status of China's medical services system and tourism development system in 2012 and 2019 by using trend surface analysis and the degree of coupling coordination as the observation. Let (*X*_*i*_, *Y*_*i*_) be the spatial location of the *i*th province and *Z*_*i*_(*X*_*i*_, *Y*_*i*_) be the trend function of the *i*th province, where the *X*-axis represents the east–west direction, and the *Y*-axis denotes the north–south direction.

### Spatial Autocorrelation

Spatial autocorrelation is a description of the degree of similarity and spatial association between the attribute values of spatially adjacent regional units and is used to reveal the spatial interactions of a geographical phenomenon between contiguous territories (73, 74).

In this work, global spatial correlation is measured using Moran's *I*, which takes the values in the range [−1,1]. The closer Moran's *I* is to 1, the stronger the degree of agglomeration, and the closer it is to −1, the stronger the degree of dispersion. An index of <0 indicates a negative correlation; and that >0 denotes a positive correlation. A random distribution is signified as it equals to 0; and a result suggesting 1 or −1 demonstrates an extremely strong positive or negative correlation (74). The specific formula for the global Moran's *I* is as follows (39):


(5) $$\begin{array}{r} I=\frac{n\sum_{i=1}^{n}\sum_{j=1}^{n}\omega_{i,j}\left(x_{i}-\bar{x}\right)\left(x_{j}-\bar{x}\right)}{\sum_{i=1}^{n}\sum_{j=1}^{n}\omega_{i,j}\sum_{i=1}^{n}{\left(x_{i}-\bar{x}\right)}^{2}} \end{array}$$

where *n* is the number of observations of variable *x*; *x*_*i*_ *and x*_*j*_ are the observations of variable *x* at positions *i* and *j*, respectively; $\bar{x}$ is the mean of all observations; and ω_*i, j*_ is the spatial weight matrix value.

We also identify high- and low-value agglomerations of the coupled coordinated development across provinces, that is, hot and cold spots to observe local characteristics, on the basis of the Getis-Ord Gi^*^ statistic in ArcGIS to avoid global spatial autocorrelation that may mask local spatial heterogeneity (75).

### Geodetector

Geodetector is a set of statistical methods that detect spatial variability and reveal the driving forces behind it. It can detect the extent to which a factor explains the spatial variation of the dependent variable and reveal the source of spatial variation in the dependent variable (76). Moreover, it is widely used in nature and society because it does not require the assumption of linearity, and it has become a master method in geography to detect the mechanisms of regionalism and variability. The model expresses as follows:


(6) $$\begin{array}{r} q=1-\frac{1}{N\sigma^{2}}\overset{L}{\underset{h=1}{\sum }}N_{h}\sigma_{h}^{2}\; \end{array}$$

where *h* is the stratification of the variable or factor; *L* is the number of stratification of the variable or factor; *N* and *N*_*h*_ are the number of cells of the full region and stratification h, respectively; σ^2^and $\sigma_{h}^{2}$ are the variance of the full region and stratification *h*, respectively.

The value of *q* is used to measure the degree of explanation of the spatial variance of the detection factor *X* on the attribute *Y*. The value of *q* ϵ [0, 1]. A larger ***q*** value indicates a greater influence of the detection factor on the variable, and the stronger the heterogeneity. When ***q*** = 0, the detection factor has the weakest explanatory power on the variables, and there is no spatial heterogeneity; when ***q*** = 1, the detection factor has the greatest explanatory power on the variables and has high spatial heterogeneity.

## Results

### Results of the Comprehensive Levels

**Figure 2:**
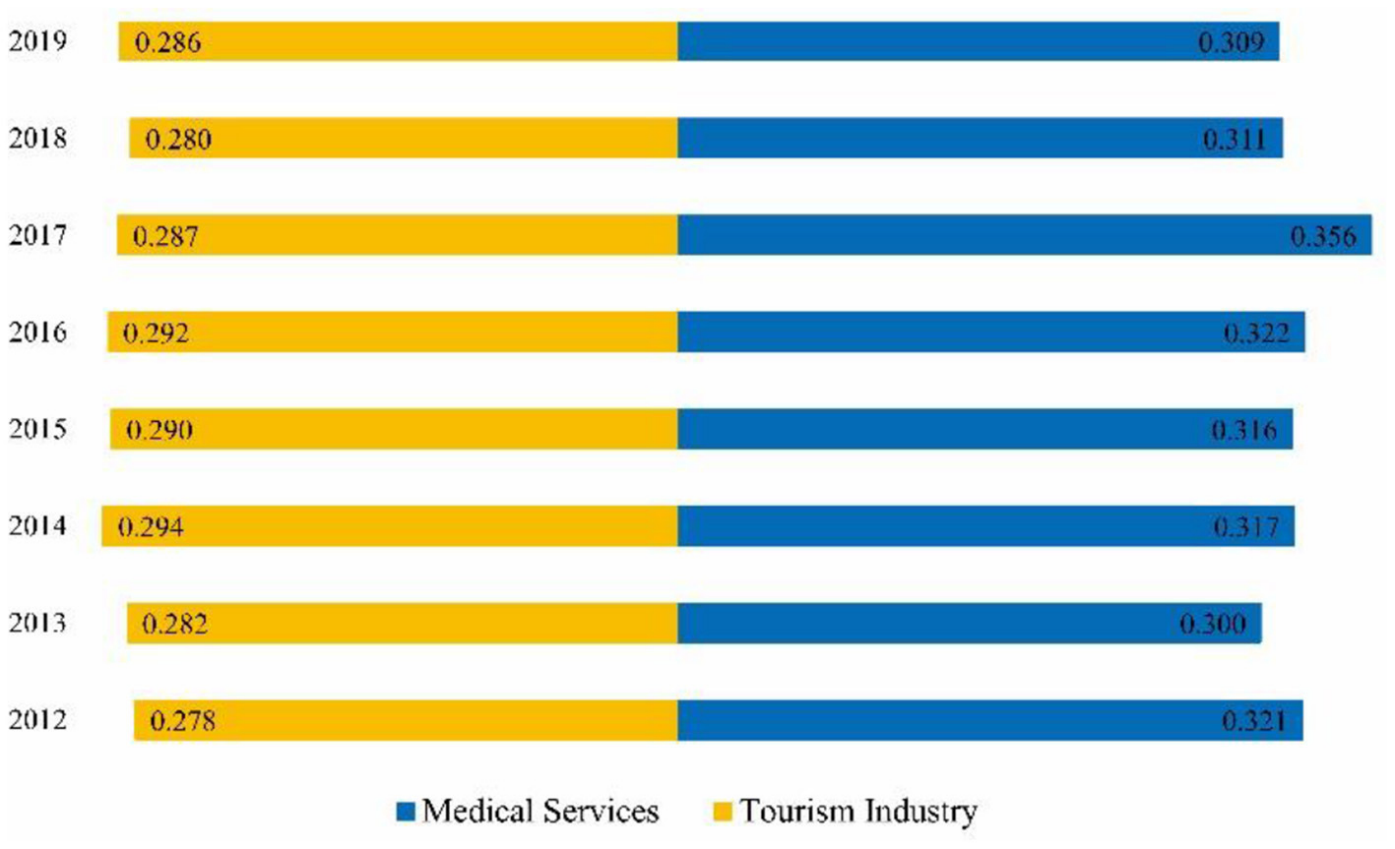
Trends in the comprehensive levels of tourism development and medical services.

A comprehensive level index of China's tourism development system and medical services system from 2012 to 2019 was calculated using Equation (1) (Figure 2). Overall, the development level of tourism development and medical services in China showed a slight upward trend in fluctuation. The results of the tourism development comprehensive measure indicate that the development level index of tourism development increased from 0.278 in 2012 to 0.286 in 2019. Meanwhile, the development level index was >0.290 from 2014 to 2016, with an average annual growth rate of 3.1%. This result indicates that China's tourism industry has undergone a slow upward trend during this period.

The results of the comprehensive measure of medical services demonstrated that the development level index increased from 0.309 in 2012 to 0.321 in 2019. In particular, the average annual growth rate of 9.1% for 2013–2017 indicates that China's medical services industry has developed at a fast rate during this period. The comparison of the index of tourism development and medical services indicated that the development trend of tourism development and medical services in China was the same from 2012 to 2019. However, differences were also observed. The level of medical services development becomes much better than tourism development as the gap between the index of tourism development and medical services increases. During these 5 years, the income of residents has increased as China has built a moderately prosperous society in an all-round way, and the paid leave system has been gradually implemented. China's tourism has shifted from the development model of attraction tourism to the development model of holistic tourism (40). However, the pace of tourism development has slowed down compared with that of the previous decade. China is the world's most populous country, and its large population base and rapidly growing aging population bring about a continuously growing demand for healthcare services; accordingly, the healthcare service industry is developing at a faster rate (30). Therefore, some differences can be observed between the level of medical service and tourism development.

### Results of the Degree of Coupling Coordination

The degree of coupling coordination of the tourism development system and medical services system from 2012 to 2019 was calculated on the basis of Equations (2)–(4). Then, the coordination development stages were divided (Table 3). During this period, the degree of coupling slightly changed, and the average value is >0.9. The change in the degree of coordination and the coupling coordination is not significant. The above data show that although the overall coupling and coordination between tourism development and medical services show a slight increasing trend, it is only at the stage of barely balanced development. Moreover, the level of coupling coordination between the two aspects requires further improvement. According to the comparison of the comprehensive development level, coordination and interaction can be divided into three types. If *q*_1_ > *q*_2_, then medical services are lagging; if *q*_1_ > *q*_2_, then tourism development and medical services are simultaneously developing; if *q*_1_ < *q*_2_, then tourism development is lagging. Table 3 shows that tourism development was always lagging behind from 2012 to 2019.

**Table 3:** Coupling coordination degree and the development stage between tourism development and medical services.

**Year**	**C**	**T**	**D**	**Coupled and coordinated development phase**
2012	0.945	0.300	0.520	Barely balanced development with TD lagged
2013	0.952	0.291	0.513	Barely balanced development with TD lagged
2014	0.950	0.306	0.526	Barely balanced development with TD lagged
2015	0.954	0.303	0.525	Barely balanced development with TD lagged
2016	0.953	0.307	0.529	Barely balanced development with TD lagged
2017	0.948	0.322	0.539	Barely balanced development with TD lagged
2018	0.954	0.296	0.518	Barely balanced development with TD lagged
2019	0.961	0.297	0.520	Barely balanced development with TD lagged

**Figure 3:**
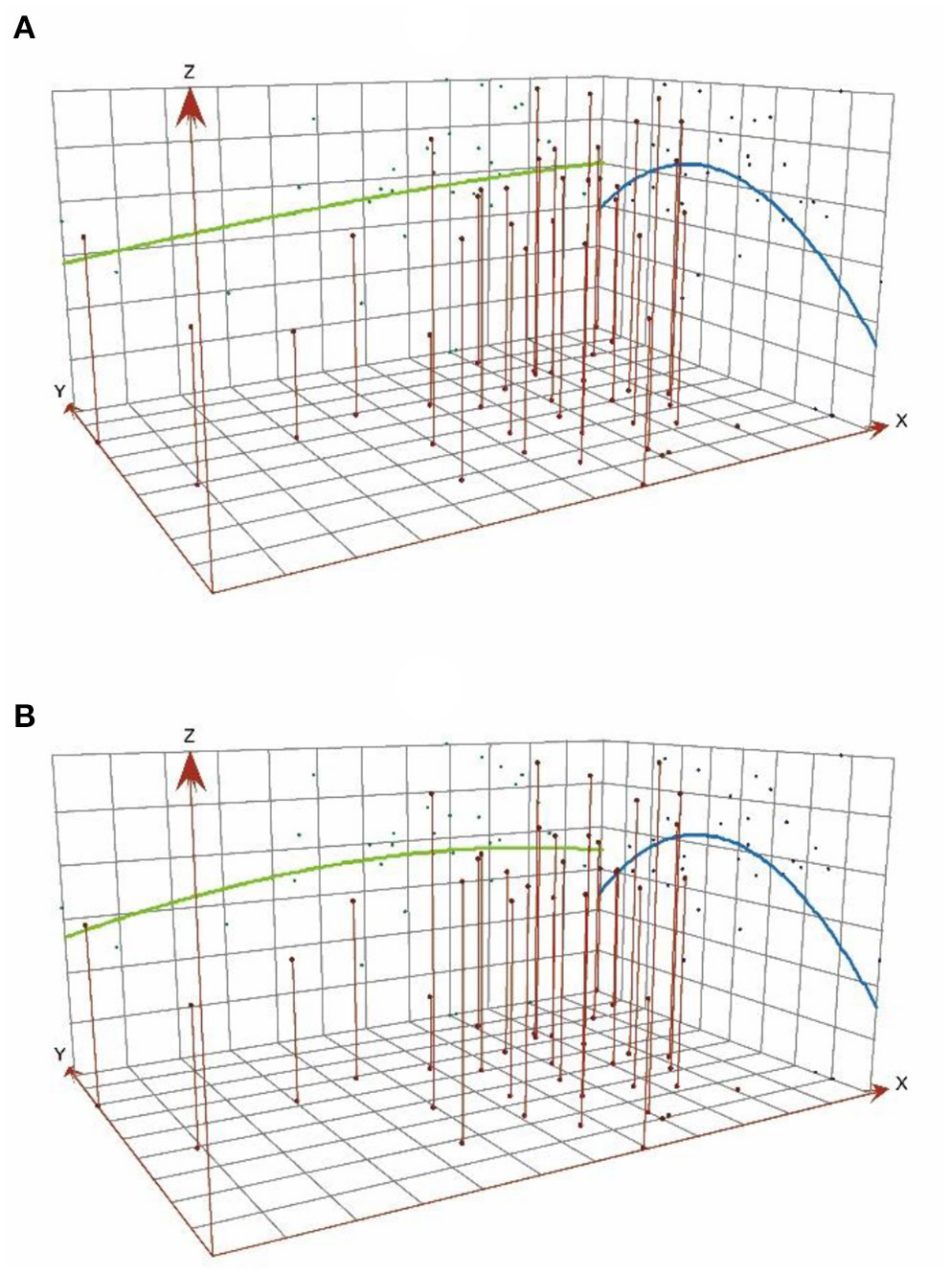
Analysis of spatial trends in the coupled coordination of tourism development and medical services **(A)** 2012, **(B)** 2019.

A spatial trend analysis was conducted on the basis of ESDA technology to further reveal the state of coupling coordination between tourism development and medical services in different regions of China. Figure 3 shows the results. The overall spatial pattern of the degree of coupling coordination is “high in the north and low in the south, high in the east and low in the west.” In the figure, the *X*-axis is the east direction, the *Y*-axis is the north direction, and the *Z*-axis represents the degree of coupling coordination of each province. The blue curve is the best-fit curve of the coupling coordination degree in the north–south direction, and the green curve is the best-fit curve of the coupling coordination degree in the east–west direction.

From the characteristics of the curve change, the north–south direction shows a clear spatial distribution of “high in the middle and low on both sides, and high in the north and low in the south.” The provinces with high values of coupling coordination include Shandong, Beijing, Zhejiang, Jiangsu, and Shanghai, most of which are located in the coastal area in the north of China. Beijing is unique because it is the national capital. The other provinces are located near the coast. Their high level of development of medical services and tourism and their high socio-economic conditions provide a good basis for the coupling and coordination compared with the inland. By contrast, the low-value coupling coordination zone is located mainly in the provinces of Anhui, Yunnan, Guangxi, Fujian, and Hainan, most of which are located in the south of China. From the socio-economic point of view, these provinces are much less developed than the northern coastal areas, thus having a lower degree of coupling coordination. The feature in the north–south direction remains stable over time and has not significantly changed. The trend in the east–west direction is more active, evolving from a “high in the east and low in the west” to an inverted “U” shape. However, the curve always maintains the characteristic in which the east is higher than the west. The gap between the east and the west ends is slightly moderated, indicating that the gap between the coupling of tourism development and medical services in China's provinces is gradually decreasing in the east–west direction, and the inter-regional coordination improved to a certain extent.

The reasons for this result are inseparable from the geographical location and economic and social conditions. In the east–west direction, the provinces of Ningxia, Tibet, and Qinghai in the western region of China have a harsh ecological environment, making it difficult to develop tourism (38). The regions located in the inland northwest have a low level of economic development and inadequate health care infrastructure. By contrast, the eastern provinces have convenient transportation, pleasant climate, and many international cities with relatively high socioeconomic and cultural levels, providing a good foundation for medical services and tourism development (51). Although the north also has a certain tourism base and a high level of urbanization, the medical environment is not comfortable compared with the south. The south has a warm and humid climate with high forest vegetation cover and a sound healthcare infrastructure. Thus, the overall level of development coordination is higher than that in the north.

### Spatial Pattern and Development Characteristics

Moran's *I* and its related indicators for the coupling coordination degree of tourism development and medical services in China from 2012 to 2019 were calculated by using Equation (5) to test the geospatial correlation of the coupling coordination degree, as shown in Table 4. The results show that Moran's *I* was positive in the period 2012–2016, ranging from 0.120 to 0.132, and it passed the 5% significance test level (77). This notion indicates that there is a significant spatial clustering trend in the coupling coordination degree over this period, with a “proximity dependency” effect between geographically adjacent provinces. The Moran's *I* and Z-value gradually decreased with *p* > 0.05 between 2017 and 2019. The spatial pattern of the coupling coordination degree transformed from clustered distribution to random distribution. The overall development level of tourism development and medical services in China was improved, and the development was balanced.

**Table 4:** Global autocorrelation analysis of the coupling coordination degree of tourism development and medical services.

**Year**	**Moran's *I***	***Z*-value**	***p*-value**	**Spatial pattern**
2012	0.120	1.999	0.046	Clustered distribution
2013	0.128	2.104	0.035	Clustered distribution
2014	0.121	2.014	0.044	Clustered distribution
2015	0.108	1.849	0.045	Clustered distribution
2016	0.132	2.162	0.031	Clustered distribution
2017	0.073	1.383	0.167	Random distribution
2018	0.089	1.583	0.114	Random distribution
2019	0.074	1.383	0.167	Random distribution

**Figure 4:**
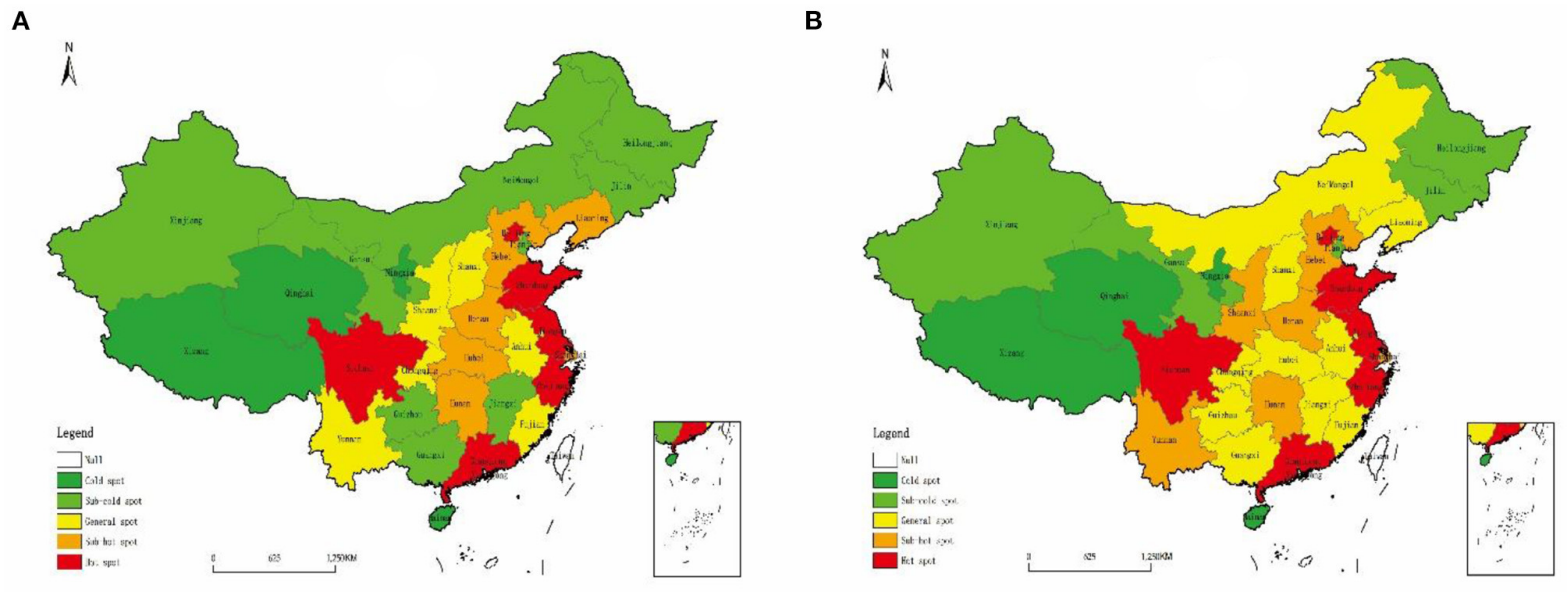
Cold–hot spots of the degree of coupling coordination of tourism development and medical services **(A)** 2012, **(B)** 2019.

The Getis-Ord Gi^*^ statistics was used to map the spatial and temporal distribution of cold–hot spots in the coupling coordination degree of tourism development and medical services in China in 2012 and 2019 and further explore the local spatial pattern (Figure 4). The hot and cold spot areas do not greatly change. By contrast, the sub-hot, sub-cold, and general areas evidently change. This result indicates an overall spatial development pattern of “cold in the northwest and hot in the southeast,” with an obvious gradient distribution pattern.

The hot spot areas are concentrated, with the radiation range roughly showing a strip-like distribution along the eastern coast. The cold spot areas are scattered but concentrated in the west with a clump-like distribution. The sub-hot spot areas distributed from a strip pattern extending from the center to the north to a sporadic point pattern. The general area shows a group-loading distribution and has increased by four provinces from 2012 to 2019. The spatial pattern of the sub-hotspot area shows a strip-like distribution in the north and a group-packed distribution in the south. Then, the pattern shows a spatial distribution trend of decreasing in the north and expanding in the south. These results show significant regional differences in the degree of coupling coordination of tourism development and medical services in China. The regions with a high overall level of coordinated development are concentrated mainly in provinces with good health care conditions or developed tourism industry. Furthermore, there may be a link between this distribution characteristic and the degree of economic development of each province.

### Driving Mechanism of Coupling Coordination

The coordinated development of tourism development and medical services is a more complex process driven by a variety of factors, such as natural, economic, policy, and accessibility. To verify the results of the previous analysis and considering the availability of data, the factors, such as economic development level, industry structure, transportation conditions, ecology, urbanization level, and government regulation ability, can be combined with the results of other scholars' studies. Accordingly, an aggregated index system consisting of six aspects and 10 detection factors was formulated based on literature and considering the data availability (Table 5) (27, 38, 41, 53, 78). ArcGIS software was used to classify the natural break points of each factor, and the driving mechanism of coupling coordination between tourism development and medical services was detected and analyzed according to the geodetector.

**Table 5:** Driving factors affecting coupling and coordination between tourism development and medical services (*q* value).

**Driving mechanism**	**Detection factors**	**2012**	**2013**	**2014**	**2015**	**2016**	**2017**	**2018**	**2019**	**Mean**
Economic development level	Per capita GDP (yuan)	0.290	0.292	0.269	0.255	0.288	0.250	0.268	0.261	0.272
	Per capita disposable income (yuan)	0.348	0.354	0.354	0.311	0.384	0.214	0.331	0.277	0.322
Industry structure	Proportion of tertiary industry (%)	0.099	0.086	0.090	0.334	0.282	0.073	0.166	0.263	0.174
Transportation conditions	Total length of highways (km)	0.279	0.279	0.177	0.246	0.178	0.243	0.168	0.195	0.221
	Length of railways in operation (km)	0.133	0.140	0.111	0.103	0.108	0.157	0.081	0.105	0.117
Ecology	Forest coverage rate (%)	0.126	0.125	0.128	0.124	0.137	0.166	0.097	0.150	0.132
	Per capita area of park green land (*m*^2^)	0.032	0.075	0.129	0.086	0.104	0.081	0.146	0.330	0.123
Urbanization level	Proportion of urban population (%)	0.310	0.304	0.215	0.244	0.280	0.151	0.198	0.218	0.240
Government regulation ability	Per capita budgetary expenditure (yuan/per person)	0.254	0.280	0.262	0.137	0.319	0.363	0.363	0.345	0.290
	Total investment in fixed assets (100 million yuan)	0.734	0.729	0.723	0.698	0.680	0.599	0.645	0.221	0.629

According to Table 5, the differences in the influence of each factor of coupling coordination of tourism development and medical services from 2012 to 2019 are more obvious. First, the power of the government to regulate is always an important factor influencing the coordinated development. In particular, total investment in fixed assets has the greatest influence with its mean value of 0.629, but its influence decreases year by year. Then, the level of economic development and urbanization also have a large influence. Other factors also have different degrees of influence on the degree of coordination in different years. The specific influencing mechanisms are as follows.

(1) Economic development level. The *q*-values of per capita GDP and per capita disposable income are >0.2, indicating that economic development level has a strong driving effect on the integration of tourism development and medical services. The large amount of capital investment helps the infrastructure construction of tourism and medical services development and strengthens the competitive advantage of the industry. The overall consumption level of residents also rises, which is conducive to promoting the scale of tourism and medical services demand, helping tourism and medical services and other industrial factors to achieve the agglomeration effect.

(2) Industrial structure. The *q*-value of proportion of tertiary industry varies from 0.073 to 0.334. Given that tourism and medical services belong to the category of tertiary industry, the general environment of the development of tertiary industry has a certain influence on their integration and development.

(3) Transportation conditions. According to the results of the geodetector analysis, the mean values of *q*-value of the total length of highways and railways in operation are 0.221 and 0.117, respectively. Overall, transportation is the most important factor affecting the coordination development of tourism and medical services. However, the total length of highways has a greater effect. From the overall situation in China, the railways as the external transportation facilities between regions have been relatively well-developed, but the unbalanced between regions. Therefore, it is the basic condition that affects the coordination development of tourism development and medical services.

(4) Ecology. The *q*-value of forest coverage rate shows a small continuous increase, and the per capita area of park green land is fluctuating in an upward trend. This result indicates that the sustainability of the ecological environment of the whole region is more important for the coordinated development of tourism development and medical services than the urban environment.

(5) Urbanization level. The mean *q-*value for the proportion of urban population is >0.2, indicating that the level of urbanization plays a significant role in the development of tourism and medical services, and it is a prerequisite for their coupled and coordinated development. The higher the level of urbanization, the stronger the service industry's reception capacity.

(6) Government regulation ability. Government regulation is an important manifestation of government action on tourism development and medical services development, and it is an external guarantee force to promote the coordinated development of both. Table 5 demonstrates that the influencing value of per capita budgetary expenditure increases year by year, and the average value of *q* for total investment in fixed assets is >0.6, indicating that the government regulation ability is increasingly important to promote the coordinated development of tourism development and medical services.

## Discussion

### Temporal Dynamic Changes

The coupled and coordinated development of tourism development and medical services is a dynamic process. The coordinated development of the two aspects has an important role in promoting the sustainable development of medical tourism. The study results show that the trends in the overall development of tourism development and medical services in China are consistent, and both are on the rise. However, the gap between the two aspects is increasing, with medical services developing at a faster rate than tourism. Moreover, the coupling of tourism development and medical services systems is only at the barely balanced development stage with tourism lagging behind. This finding suggests that the development of tourism development and medical services in China is uneven. China is the world's most populous country, and its large population base and rapidly growing aging population have created a growing demand for medical services (62). The healthcare service industry is not only an important part of the healthcare sector but also a new growth area for the modern service industry because it is closely related to national strategies (30). Accordingly, China's healthcare service industry is in a golden period of rapid development. In contrast with the development of tourism, thanks to the steady growth in the income of urban and rural residents and the accelerated development of tourism transport infrastructure, such as highways and high-speed railways (40), tourism has also developed to a certain extent, but at a slower pace. Considering this gap, rapid development of health services can influence tourism and cause a range of social problems (36, 37). Therefore, paying attention to the balanced development of both aspects at different historical stages is important to improve the level of coupling and coordination and promote the sustainable development of both systems. In addition, the study results are objective and credible and help in extensively understanding the complex relationship between tourism development and medical services, providing a theoretical basis for measuring the coupled coordination of tourism development and medical services in other countries. The findings help governments guide the development of medical tourism practices and implement sustainable development strategies (15, 18).

### Spatial Dynamic Changes

In terms of spatial patterns, the coupled coordination values of tourism development and medical services in China are higher in the north provinces than in the south and higher in the east than in the west. However, the coordination between regions has improved to a certain extent, with the north–south direction being more stable and the gap between the east and west being gradually reduced. This change is a clear indication that the Chinese government's development strategy of “promoting the development of the west, the comprehensive revitalization of the northeast, the rise of the central region, and the pioneering development of the east” has achieved significant results (79). The hotspots and sub-hotspots of the coupling of tourism and healthcare services in China are basically distributed along the eastern coast in a strip-like pattern, with a gradient distribution pattern from the coast to the interior (51). However, the CCD of tourism development and medical services in China has varying spatial distribution patterns at different stages. There is a significant spatial dependence of CCD with a clear clustering effect in 2012–2016, and provinces with high (or low) CCD are spatially adjacent to each other (39). The CCD correlation was not significant in 2017–2019, tending to a random distribution with no similarity.

The above results may be related to a number of influencing factors. First, the vast size of China and the large differences in natural conditions and socio-economic development levels among provinces resulted in inconsistencies in the level of development of tourism and healthcare services, which ultimately lead to the two systems being uncoordinated. Therefore, paying attention to inter-regional coordination and reducing regional differences are important. Second, macro policies are important drivers to improve the level of coordination between tourism development and medical services (44, 80). Moreover, in medical services, local governments need to take advantage of local conditions and policies to encourage tourism and medical services to go hand in hand. Finally, the relationship between tourism development and medical services can be described as an interaction (20, 21). Tourism promotes the development of medical services, medical services support the development of tourism, and the two complement each other in a dynamic process (9, 14, 35). Therefore, cities that need to vigorously develop medical tourism can enjoy the benefits of coordinated development of tourism development and medical services, avoiding the negative effects of external shocks and maintaining sustainability (53).

## Conclusions and Implications

This work constructs an evaluation index system and uses IDLEM, CCDM, trend surface analysis, and spatial autocorrelation analysis methods in geography to study the relationship of coupling coordination between tourism development and medical services in China from 2012 to 2019. Furthermore, the dynamic change mechanism and spatial distribution of the coupling coordination between tourism development and medical service are explored. The main findings are as follows.

(1) The comprehensive level of tourism development and medical services in China has shown a year-on-year increase, and the two systems are developing in the same way. However, the gap between them is increasing, and medical services are developing faster than tourism development.

(2) The coupling coordination degree shows a good upward trend. However, with only the barely balanced development tourism development lagging behind the stage, the level of coupling coordination needs to be improved. The overall spatial pattern of the coupling coordination degree is “high in the north and low in the south, high in the east and low in the west,” with a more obvious trend in the east-west direction.

(3) From a global perspective, the coupling coordination degree in tourism development and medical services has a significant spatial clustering trend, but it gradually evolves into a random distribution pattern over time. In terms of local spatial patterns, an overall spatial development pattern of “cold in the northwest and hot in the southeast” was observed, with a clear pattern of gradient distribution.

(4) The analysis of the driving mechanism of the coupled tourism development and medical services found that the power of the government to regulate is always an important factor influencing the coordination development. Meanwhile, the level of economic development and urbanization also has a strong influence. The other factors have different degrees of influence on the coupling coordination degree in different years.

### Implications

In a theoretical sense, a comprehensive evaluation index system for the coupling coordination of tourism development and medical services in China has been constructed and quantitatively studied. This method can be used to explore the degree of coupling coordination of tourism development and medical services in different countries. This method can be extended to multiple systems, which is conducive to promoting the benign development of multiple systems.

The study findings have practical implications for the promotion of medical tourism in China. First, the Chinese government has attached great importance to the development of medical tourism in recent years, and the results can provide the government with implications for focusing on developing advantageous regions and driving weak regions. Second, the findings can provide a reference for the government to formulate relevant policies to promote the deep integration of medical services and tourism development, fully explore Chinese medicine resources, and transform them into tourism products to optimize investment. Third, enhancing cooperation between healthcare providers and tourism businesses is conducive, especially in the private healthcare services sector. For example, travel agencies work with hospitals or clinics to offer medical and tourism packages to tourists. Hotel providers and herbal medicine companies collaborate to offer a variety of medicinal bath products. Cooperation between healthcare providers and tourism enterprises can build good partnerships and increase profitability and competitiveness.

### Limitations

Although this work has established a comprehensive indicator system to study the relationship of coupled coordination between tourism development and medical services, using the 31 provinces of China as the study area, it has limitations. First, no similar studies have been conducted in other countries on the coupled coordination model of tourism development and medical services proposed, making comparisons on the same evaluation criteria considering the different national contexts of each country difficult. Second, this study starts at the macro-level of the province, which has implications for grasping the coupled coordinated development of tourism development and medical services in China. If we could start at the micro-level, such as the municipal and county levels, the data would be more accurate, and the conclusions drawn would be more reasonable and scientific. Finally, the research data are derived from existing government statistics, which are not updated on a timely basis and do not allow for timely updating of data to draw the most up-to-date conclusions.

## Data Availability Statement

The original contributions presented in the study are included in the article/supplementary material, further inquiries can be directed to the corresponding author.

## Author Contributions

SX: conceptualization, methodology, software, writing—original draft, and writing—review and editing. YZ: conceptualization, methodology, and writing—original draft. RL: writing—review and editing and supervision. MZ: supervision, conceptualization, and writing—original draft. JH: software and writing—original draft. GL and JM: conceptualization and writing—original draft. All authors contributed to the article and approved the submitted version.

## Conflict of Interest

The authors declare that the research was conducted in the absence of any commercial or financial relationships that could be construed as a potential conflict of interest.

## Publisher's Note

All claims expressed in this article are solely those of the authors and do not necessarily represent those of their affiliated organizations, or those of the publisher, the editors and the reviewers. Any product that may be evaluated in this article, or claim that may be made by its manufacturer, is not guaranteed or endorsed by the publisher.
